# Open source inert gas glove box

**DOI:** 10.1016/j.ohx.2025.e00702

**Published:** 2025-09-19

**Authors:** Mottaghi Maryam, M. Pearce Joshua

**Affiliations:** a Department of Mechanical and Materials Engineering, Western University, London, Canada; b Department of Electrical and Computer Engineering, Ivey Business School, Western University, London, Canada

**Keywords:** Glove box, 3D printing, Environment safety, Environmental control, Open hardware, Inert gas

## Abstract

A glove box is a controlled environment used for a wide range of scientific experiments. While glove boxes provide significant advantages, their high economic costs ranging from over $1,000 to over $15,000 limits their accessibility in under-resourced labs. There are lower-cost DIY designs available on the internet, but they have not been well characterized nor validated. To overcome these limitations, in this study, an open source glove box design is developed for scientific applications using readily available components and digital distributed manufacturing using open-source RepRap-class 3D printers. The ability of the glove box to hold an inert atmosphere is quantified using an oxygen analyzer. The open source glove box can be customized to the dimensions of the user and the volume of the experiment. The design also enables the use of customizable transfer chambers that can be adjusted based on the scientific application. The open source glove box is built from a low-cost enclosure while preventing contamination. The highly portable device can reduce oxygen down to 19 ppm using an inert gas. The economic savings of the validated device compared to proprietary systems is over 95 %.

## Graphical abstract

**Figure:**
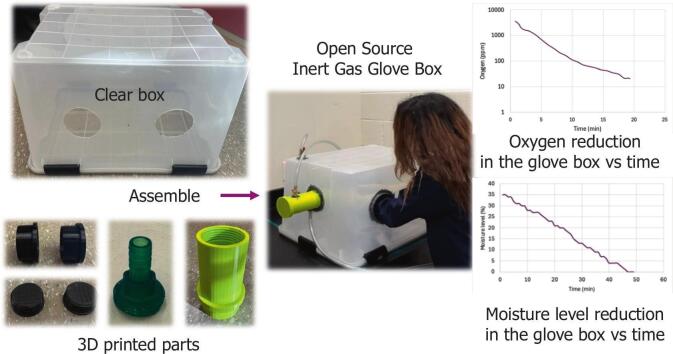

Specifications table

**Table:** 

Hardware name	*Open Source Inert Gas Glove Box*
Subject area	• Engineering and materials science • Chemistry and biochemistry
Hardware type	• Mechanical engineering and materials science
Closest commercial analog	Scienceware H50028-2001 Portable Glove Box; 86.6 L, Neoprene Gloves, 2 Gas Ports ($3,072.02CAD) *https://www.coleparmer.ca/i/scienceware-h50028-2001-portable-glove-box-86-6-l-neoprene-gloves-2-gas-ports/3477002*
Open source license	*Designs:* GNU GPL v3 Hardware: CERN OHLv2-S
Cost of hardware	CAD$87.51
Source file repository	doi:10.17605/OSF.IO/XNQK3
OSHWA certification UID	*CA000056*

## 1 Hardware in context

A glove box is a controlled environment used during chemical synthesis, materials handling and biological processes to handle sensitive materials and protect them from exposure to air or moisture [1]. It generally consists of a sealed container with two flexible gloves, a transfer chamber for sample loading and unloading, a gas supply for inter gases such as nitrogen or argon, a gas inlet hose barb, and a gas outlet ball valve [2].

Glove boxes are widely used in various scientific fields. In chemical research, they are used to handle air-sensitive and moisture-sensitive reagents or catalysts which are crucial for many synthesis methods [3]. For example, when working with metal alkyls and aryls, it is necessary to maintain oxygen levels below 15 ppm [[4], [5], [6]]. In the synthesis of tin oxide sheets, glove boxes maintain oxygen levels between 10 and 100 [7]. In the analysis of the organic solar cells, an environment with 20 ppm oxygen level should be provided [8].

In biochemical laboratories, glove boxes are essential for working with oxygen-sensitive enzymes and compounds to maintain their stability and activity [9]. In biological research, they provide sterile conditions necessary for applications like cell research [10]. Additionally, in cryo-TEM experiments, glove boxes help prevent sample dehydration and preserve the original state of specimens [4]. In nuclear laboratories, they are used to secure safe handling of radioactive materials [2]. Moreover, in battery assembly, glove boxes prevent environmental contamination and provide the safe handling of reactive materials [10], similar to the function of a fume hood in preventing the release of unsafe particles and chemicals into the environment [11].

While glove boxes provide significant advantages, their high economic costs ranging from CAD$1,424 to CAD$15,120 (see Table 1), can limit their accessibility in under-resourced labs. To address this issue there are three main approaches. First, some researchers have explored alternative approaches to conduct their targeted research. For instance, Kubota et al. adopted mechanochemistry for C-N cross-linking reactions in the solid-state to avoid the need for a glove box [1,12]. Similarly, Yaghoobi et al. employed a crystal engineering approach and optimized deposition methods to fabricate perovskite solar cells and modules without using a glove box [13]. Although it is possible to engineer experiments to avoid using glove boxes, this is not always possible. Researchers in various fields, such as material sciences, pharmaceuticals, agrochemicals, and sensors, may face challenges in the usability of synthetic methods due to not having access to a glove box [3].

**Table 1:** Representative selection of commercial glove boxes specifications.

**Commercial Proprietary Product**	**Cost (CAD$)**	**Dimensions**	**Material**	**Applications**	**Unique specifications**
sidENTRY Glove Box [14]	5,429.00	30 x 24 x 24 in	Acrylic	Medical and Laboratory	Portable
Scienceware portable glove box [15]	2,370.00	27 x 13 x 22 in	Acrylic	Isolating materials that need to be processed	Portable
Plas-Labs compact glove box [16]	15,120.00	63 x 31 x 34 in	N/A	Toxic materials and pharmaceuticals	−Determining the dry weight or moisture content of aqueous solutions, adhesives, cereals, toxic chemicals, pharmaceuticals, paper, plastics, and light radioactive materials −Multiple electrical outlet strip
Pasaurina Glove Box [17]	1,423.88	23.6 × 19.7 × 20.5 in	Body framework/Stainless steel, Positive side window portion/PC	N/A	N/A

To address the expenses associated with necessary research equipment, a second strategy involves building the equipment. There are several Do It Yourself (DIY) designs available all of which are made from a clear plastic box and elbow-length waterproof gloves. For instance, in a prominent design published by *Make Magazine* [18], PVC pipes are used for the arm holders which are securely attached to the box with sealant to prevent the leakage [19]. To manage the internal atmosphere, a ventilation fan and air hose are used to blow air out of the box which creates a slightly lower pressure inside compared to the outside. This setup helps maintain a controlled environment. This glove box is designed to be easy to use, portable, and low-cost; however, there are limitations including the lack of a transfer chamber, which restricts the ability to safely insert and remove materials without contaminating the internal atmosphere. Additionally, the atmosphere inside the box is not inert, which is critical for many laboratory applications. The method of purging oxygen using a fan is not practical for achieving the low oxygen level that is required in many scientific applications. Furthermore, the glove box has not been validated which makes it unreliable for precise laboratory research [19]. In another glove box design, PVC pipes were used as arm holders, and argon gas was introduced at 10 cubic feet per minute, which required 6.5 min to fill the box and displace the air. While the box slowed lithium metal oxidation, the presence of residual air made it unreliable for precise laboratory experiments [20]. In another found DIY glove box, the Fresno Mycology Society used short piping as arm holds and applied epoxy to secure the pipes. This glove box is also easy to use and portable but it is not designed to have an inert atmosphere [21]. Another approach used plastic water outlets for replacing the air with inert atmosphere, but again this glove box has not been validated [22]. A summary of these DIY glove boxes is shown in Table 2.

**Table 2:** DIY glove boxes.

**Maker**	**Materials**	**Validation**	**Advantage**	**Disadvantage**	**Reference**
Make Magazine	Clear plastic box, PVC pipes, sealant, elbow-length waterproof gloves, ventilation fan, air hose	Not validated	Easy to use, portable, low-cost	Lacks transfer chamber, atmosphere not inert, impractical oxygen purging method, not reliable for precise laboratory work, lacks transfer chamber, not adequate arm holds for many users	[19]
MHS	Clear plastic box, PVC pipes, epoxy, hose barb, barb valve, argon tank	Inaccurately validated	Easy to use, portable, low-cost, uses argon for inert atmosphere	Residual air present, inaccurate validation with match test, unreliable for precise laboratory experiments, lacks transfer chamber, not adequate arm holds for many users	[20]
Fresno Mycology Society	Clear plastic box, short piping sections, epoxy	Not validated	Easy to use, portable, low-cost	Lacks transfer chamber, atmosphere not inert, arm holds may not be adequate for all users, unreliable for precise lab work, lacks transfer chamber, not adequate arm holds for many users	[21]
Patti	Clear plastic box, PVC pipes, PVC glue, silicone, plastic water outlets	Not validated	Easy to use, potentially useful for basic tasks	A tmosphere control uncertain, may not meet requirements for sensitive applications, lacks transfer chamber, not adequate arm holds for many users	[22]

A third approach, building on the second involves decentralized production of free and open-source hardware (FOSH) [23,24]. This approach offers cost reductions, customization opportunities, and enhanced control for scientists [[24], [25], [26], [27]]. Open-source digital manufacturing technologies, such as the self-replicating rapid prototyper (RepRap) 3-D printer [[28], [29], [30]], significantly contribute to the advancement of open hardware [31,32]. Recent literature on FOSH applications in scientific settings demonstrates average cost savings of 87 % compared to proprietary tools of equivalent or those of lesser quality [27]. By incorporating open-source electronics like Arduino technology [33], savings can increase to 89 %. The implementation of RepRap-class 3D printing can result in savings of 92 %, while combining open-source electronics and RepRap-class 3D printing can lead to savings of 94 % [27]. Scientists who engage in constructing their own hardware [[32], [33], [34]] can take advantage of parametric FOSH [[31], [32], [33], [34], [35]] to obtain high-quality customized research equipment based on free digital designs [23,[36], [37], [38]]. Existing DIY glove box designs used in the second approach do not rely on distributed digital manufacturing for parts. This is a missed opportunity as the literature indicates distributed manufacturing with 3D printing reduces costs and increase customization on a wide range of products [[39], [40], [41], [42]]. In addition, all of the designs in Table 2 have not been well characterized and validated.

To overcome these limitations, in this study, an open source glove box design is developed for scientific applications using readily available components and digital distributed manufacturing using a RepRap-class 3D printer. The ability of the glove box to hold an inert atmosphere is quantified using an oxygen analyzer. This open-source glove box design effectively bridges the gap between unvalidated DIY glove box solutions and expensive commercial systems by offering a validated, low-cost, and customizable alternative. Unlike many DIY designs, it includes a transfer chamber and has been quantitatively validated to reach oxygen levels as low as 19 ppm, which shows reliable performance for scientific applications. At the same time, it maintains the adaptability and affordability of DIY approaches through open-source digital manufacturing methods by achieving over 95 % cost savings compared to commercial models.

To attempt to enhance the inert atmosphere within the glove box, iron powder was also used as an oxygen scavenger [43]. The use of iron as an oxygen scavenger is based on its chemical reactivity with oxygen. When iron is exposed to oxygen, it undergoes an oxidation reaction that forms iron oxide (Fe_2_O_3_ or Fe_3_O_4_). This reaction can, in theory, remove oxygen from the environment. The reactions can be described by the following equations:

(1) $$4Fe+3O_{2}\to 2{Fe}_{2}O_{3}$$

(2) $$3Fe+2O_{2}\to {Fe}_{3}O_{4}$$

These exothermic reactions proceed spontaneously in the presence of oxygen which gradually reduce the oxygen content inside the sealed glove box. Also, when moisture is present in the environment, iron powder can also reduce moisture through its reaction with water. This reaction, where iron reacts with both oxygen and water to form iron hydroxide, can be represented as:

(3) $$4Fe+3O_{2}+4H_{2}O\to 4Fe{\left(OH\right)}_{3}$$

This process not only removes oxygen and moisture from the environment, but the presence of moisture accelerates the oxidation process, which makes the iron powder more efficient at scavenging oxygen and maintaining a more inert and stable atmosphere within the glove box [43].

### 1.1 Example calculation

Considering equation (1), 1 mol of Fe reacts with 0.75 mol of O_2_. The molar mass of Fe is 55.85 [44] g/mol, and of O_2_ is 32 g/mol [45]. Therefore, 1 g of Fe can scavenge:

(4) $$Moles\;of\;Fe=\frac{1\;g}{55.85\;g/mol}\approx 0.0179\;mol$$

(5) $$Moles\;of\;O_{2}\;scavenged\;=0.0179\;\times \;0.75\;\approx 0.0134\;mol$$

(6) $$Mass\;of\;O_{2}\;scavenged\;=0.0134\;\times \;32\approx 0.429\;g$$

At standard temperature and pressure (STP), 1 mol of O_2_ gas occupies 22.4 L [46]. Therefore, 0.0134 mol O_2_ occupies:

(7) 0.0134×22.4≈0.3L

This suggests that 1 g of Fe can remove about 300 mL of oxygen gas at standard conditions. In the current glove box (with the capacity of 74 Qt equal to 70.2 L), the initial oxygen concentration (∼3,520 ppm) represents:

(8) $$3,520\;ppm=\frac{3,520}{10^{6}}\times 70.2\;L\approx 0.247L\;of{\;O}_{2}\;$$

This shows that, in theory, 1 g of Fe powder could scavenge more oxygen than required to reach 0 ppm in the box. It should be noted, however, that in the present study, iron powder was observed to reduce oxygen levels only to 19 ppm. This suggests that practical factors limit this capacity. The formation of a passivation layer of iron oxide on the surface of the iron powder reduces the availability of reactive sites, which inhibit further oxygen uptake. Additionally, the lack of sufficient moisture can slow down the reaction kinetics, particularly for reactions that rely on water as a catalyst. Incomplete exposure of the iron particles to the glove box atmosphere also limits their effectiveness, as not all reactive surfaces come into contact with the gas phase.

## 2 Hardware description

The open source glove box can provide low-cost custom components since the connections are fabricated by a RepRap-class fused filament 3-D printer, and the other parts including the clamps, the gloves and the box are readily available in the market. The arm holds are customizable and can be resized based on the user convenience.

• Low-cost enclosure for preventing contamination while doing scientific experimental
• Economic savings of over 95 %
• Customizable arm holds
• Customizable transfer chambers that can be adjusted based on the user’s convenience and the scientific application.
• Portability
• Customizable volume based on the user need
• Oxygen reduction down to 19 ppm using an inert gas

## 3 Design files

### 3.1 Design files summary

All design files associated with this work are openly available in the OSF repository at https://doi.org/10.17605/OSF.IO/XNQK3.

A complete set of designs (STL and STEP) for the parts to be printed, along with videos demonstrating the validation of oxygen and moisture content and the operational process, is available on the Open Science Framework (OSF) repository [47].

**Fig. 1:**
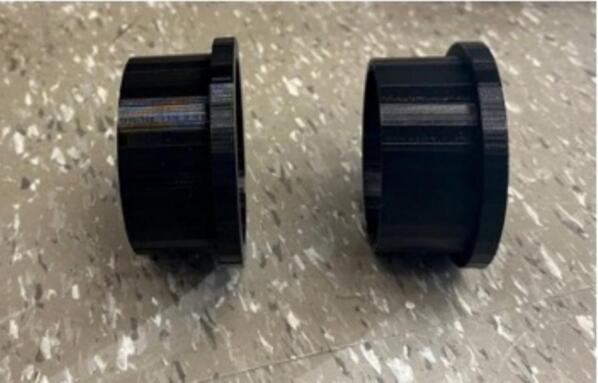
3D printed arm holds.

Arm hold that is used for placing the gloves (Fig. 1). This can be modified based on the hand size of the operator. This part can be printed with using common FFF-based 3-D printing polymers like polyethylene terephthalate glycol (PETG) or polylactic acid (PLA). Other materials are also possible like polypropylene if specific chemical compatibility [48] is necessary.

**Fig. 2:**
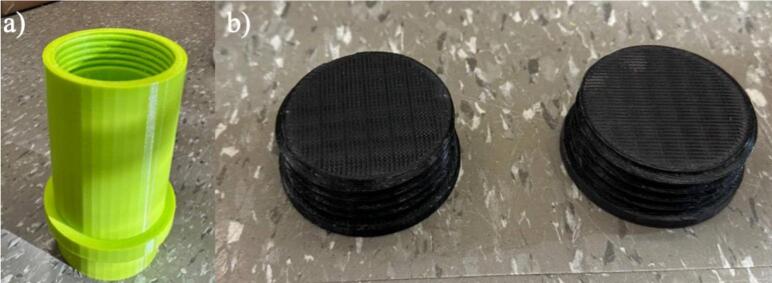
3D printed a) transfer chamber, b) transfer chamber cap.

The transfer chamber and transfer chamber cap are depicted in Fig. 2a-b, respectively. The required materials and equipment for the experiment can be placed and removed through this transfer chamber. Two holes in this transfer chamber are designed to insert a barb valve and a hose barb. After opening the cap and placing or removing the required materials into or from the transfer chamber, the transfer chamber area will be purified by purging nitrogen gas from the hose barb and exiting the gas through the barb valve. This ensures that the box does not get contaminated by opening the transfer chamber each time. Two caps are required for the transfer chamber. These parts should also be printed with PETG or PLA.

**Fig. 3:**
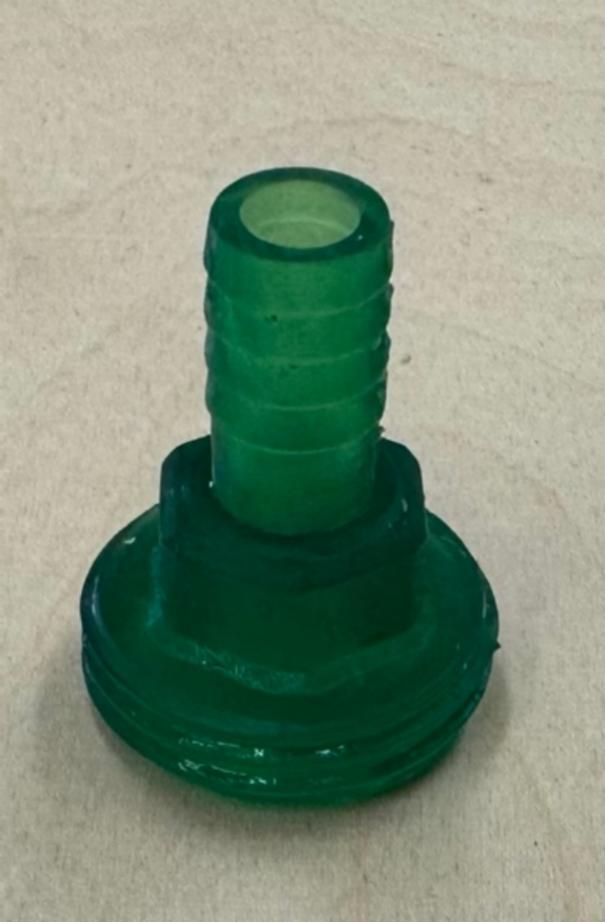
3D printed hose barb.

The hose barb is shown in Fig. 3 [49]. The inert gas (e.g. nitrogen gas) is purged to the box through this part. Two of these hose barbs are required; one for purging the gas to the box and the other one for purging the gas to the transfer chamber. This part should be printed by stereolithography (SLA) printer like the Prusa SL1S to ensure that the gas does not leak [50].

## 4 Bill of materials

### 4.1 Bill of materials summary

The clear plastic storage container serves as the main body of the glove box, which provides a transparent and enclosed workspace. Trionic gloves are attached to the arm holds that allow the safe handling of materials and execution of laboratory processes inside the glove box. Instant epoxy is used to seal any gaps and create an airtight environment. The gloves are secured to the arm holds using gear clamps to prevent any leakage. A hose barb is used to introduce inert gas into the box, while a barb valve vents the gas outside. To monitor the internal atmosphere, an oxygen analyzer is used to measure the oxygen levels inside the box and ensure a controlled and safe working environment.

The BOM of the printed parts are shown in Table 5.

**Table 3:** Design file information for the open source glove box.

**Design file name**	**File type**	**STL rendering**	**Open source license**	**Location of the file**
*arm hold*	STL, STEP		GNU GPL v3	https://osf.io/m56dz https://osf.io/v2xye
*transfer chamber*	STL, STEP		GNU GPL v3	https://osf.io/89mg6 https://osf.io/y5hde
*transfer chamber cap*	STL, STEP		GNU GPL v3	https://osf.io/34zy6 https://osf.io/rcsp2
*hose barb*	STL		GNU GPL v3	https://osf.io/bfp42
*Operation*	Video	−	GNU GPL v3	https://osf.io/ugkfw
*Oxygen Content Validation*	Video	−	GNU GPL v3	https://osf.io/arnvs
*Moisture Content Validation*	Video	−	GNU GPL v3	https://osf.io/sbnxm

**Table 4:** List of hardware to be purchased for assembly with the printed parts.

**Component**	**Number**	**Cost per unit −currency**	**Total cost −** **currency**	**Source of materials**	**Material type**
74 Qt Storage container	1	CAD$25.97	CAD$25.97	https://www.walmart.ca/en/ip/sterilite-75-l-hingelid-box-flat-gray-gray/6000203647664	Plastic
Trionic gloves	1	CAD$8.43	CAD$8.43	https://www.grainger.ca/en/product/p/MPA517316	Plastic
Instant epoxy	1	CAD$14.99	CAD$14.99	https://www.amazon.ca/LePage-Instant-Speed-Epoxy-https://doi.org/1028091/dp/B019GISEHW/ref=sr_1_4_sspa	Polymer
Gear clamps	4	CAD$3.33	CAD$9.99	https://www.amazon.ca/NEBURORA-Adjustable-Stainless-Diameter-Automotive/dp/B0BZSC47QC/	Metal
Barb valve	2	CAD$8.50	CAD$16.99	https://www.amazon.ca/Tnuocke-Fitting-Coupler-Operation-H-054–3/dp/B09FJWKC6R	Metal
3D printing resin	1	CAD$19.99 (per bottle)	CAD$0.14	https://www.amazon.ca/ELEGOO-Standard-UV-Curing-Photopolymer-Translucent/dp/B07Z9MZJGW/	Resin
3D printing filament	1	CAD$27.99 (per kg)	CAD$11	https://www.amazon.ca/Polymaker-Filament-1–75 mm-Strong-Cardboard/dp/B09DKP4TLW/	Plastic
Total			CAD$87.51

**Table 5:** BOM of 3-D printed parts.

**Part**	**Material used**	**Cost (CAD$)**
Transfer chamber	102.74 g/ 34.45 m	2.88
Arm holds	208.89 g/70.04 m	5.85
Transfer chamber cap	80.94 g/ 27.14 m	2.27
Hose barb	6.97 mL	0.14
Total	PETG: 392.57 g/71.63 m Resin: 6.97 mL	11.14

In addition to the specific glove box components listed in Table 4 and Table 5, Table 6 presents key auxiliary components required for effective operation of the glove box. These include a refillable nitrogen gas cylinder and an oxygen analyzer, which are standard equipment typically found in most laboratories utilizing glove boxes. These components are not included in the primary cost analysis of the glove box itself, as they are shared resources and not unique to the glove box design. Nonetheless, they are needed for maintaining the inert atmosphere and validating the performance of glove box.

**Table 6:** Auxiliary Laboratory Components for Glove Box Operation.

Part	Number	Approximate Market Cost
Oxygen analyzer	1	CAD$1,800
Nitrogen 99.999 % UHP T	1	CAD$50

### 4.2 Build instructions

1. The first step is 3-D printing all components listed in Table 3. The printing parameters are listed in Table 7 and Table 8. They can be printed on any RepRap-class fused filament fabrication-based 3-D printer. The 3-D printed parts shown in Fig. 1 and Fig. 2 were printed on an Original Prusa i3 MK3S & MK3S+ (Prusa Research, Prague, Czech Republic) and the part shown in Fig. 3 was printed on an Original Prusa SL1S SPEED printer.

**Table 7:** FFF printing parameters.

**Parameter**	**Amount**
Layer Height	0.18 mm
Initial Layer Height	0.425 mm
Wall Thickness	1 mm
Infill Density	20 %
Infill Line Distance	5 mm
Printing Temperature	210 °C
Build Plate Temperature	60 °C
Print Speed	60 mm/s
Infill Speed	40 mm/s
Wall Speed	30 mm/s
Travel Speed	175 mm/s
Initial layer Speed	15 mm/s
Support density	30 %

**Table 8:** SLA printing parameters.

**Parameter**	**Amount**
Layer Height	0.05 mm
Initial Layer Height	0.05 mm
Exposure Time	5 s
Initial Exposure Time	40 s
2. Next, purchase the other components listed in Table 4 (Fig. 4).

**Fig. 4:**
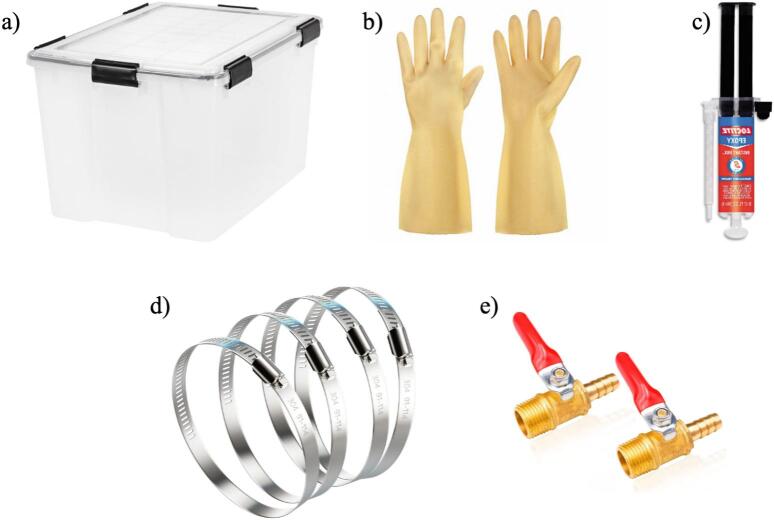
Purchased components: a) container, b) gloves, c) instant epoxy, d) clamps, e) barb valve.
3. Cut the arm hold with a rotary tool, a drill, or a cutter, transfer chamber, and wire holes on the container wall (Fig. 5).

**Fig. 5:**
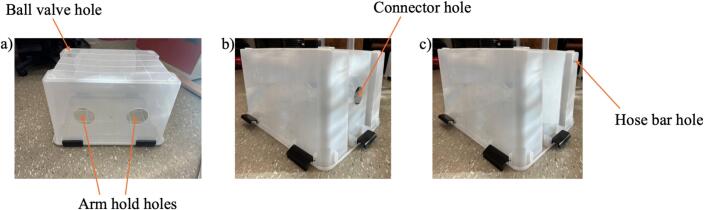
Cutting step, a) ball valve and arm hold holes, b) connector hole, and c) hose bar hole.
4. Place the arm holds, transfer chamber, barb valve, and the hose barb in the holes (Fig. 6).

**Fig. 6:**
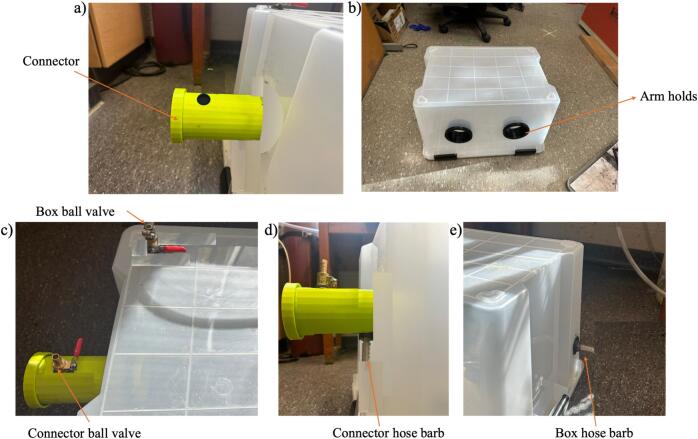
Inserting a) transfer chamber, b) arm holds, c) barb valves, d) connector hose bar, and e) box hose barb.
5. Place the gloves around the arm hold parts and fix them on their place by the gear clamps. Be careful about the orientation of the gloves as they match the left- and right-hand sides. Based on the design, the orientation of the box is upside down and the lid should be located on ground (Fig. 7).

**Fig. 7:**
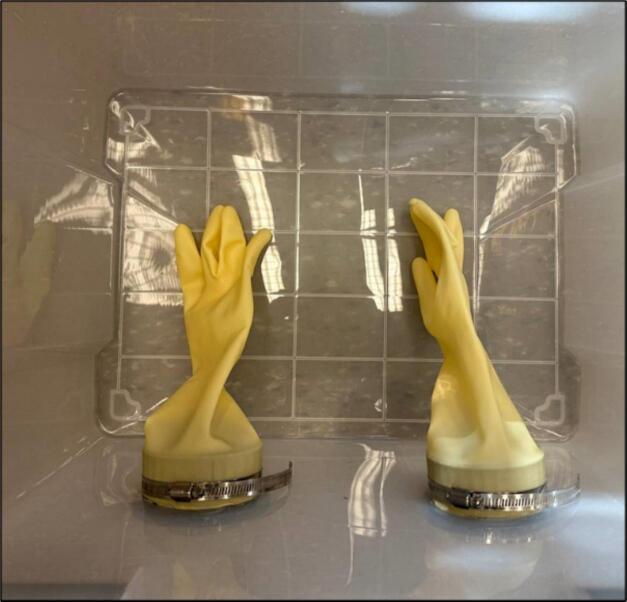
Inserting gloves.
6. Apply the instant epoxy around all the drilled holes to prevent leakage and leave it to dry overnight (Fig. 8).

**Fig. 8:**
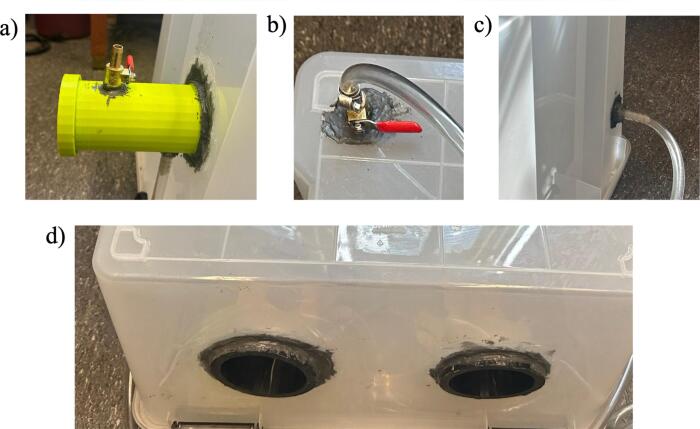
Sealing the holes of a) connector and connector ball valve, b) box ball valve, c) box hose barb, and d) arm holds, by applying instant epoxy.
7. After making sure that the epoxy is dried overnight, set up the inert gas flow, a T-junction is used to split the flow from the tank into two separate paths (Fig. 9a). Nitrogen gas is used here, but any other inert gas can be used based on related work.

**Fig. 9:**
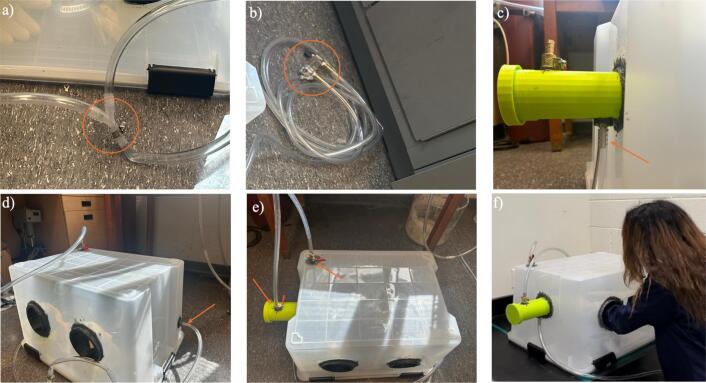
a) Inserting T-junction, b) Inserting flow meter, c) Inserting pipe to the box hose barb, d) Inserting pipe to the chamber hose barb, e) Inserting pipes to barb valves, and f) Assembled glovebox..
8. Begin by connecting the regulator to the nitrogen cylinder to control the pressure. Attach a flow meter to the regulator to monitor and set the desired flow rate (Fig. 9b).
9. Attach one pipe from the T-junction to the box hose barb and the other one to the transfer chamber hose barb (Fig. 9c and Fig. 9d).
10. For the gas exhaustion system, connect two additional pipes to barb valves (Fig. 9e). Make sure that the ends of these exhaust pipes are placed inside a hood to safely vent the nitrogen. The supplementary video is provided [47].

### 4.3 Operation instructions

The steps to operate the open source inert-gas glove box are:

1. Open the gas tank and let the gas come to the box (capacity: 74 Qt). At first, purge the gas to the tank with the flow rate of 23 L per minute to fill the box in 3–4 min [47].
2. Make sure that the cap on the outer side of the transfer chamber is closed, but on the inner side, it should be open.
3. Dilute some liquid soap and apply it with a sponge on the hole areas and anywhere there is a possibility of a gap. If bubbles are observed around the holes, apply instant epoxy to those areas again.
4. After ensuring that the box is sealed, close the barb valves and purge nitrogen into the box at a flow rate of about 4.7 L per minute. In this way, the box will be filled with nitrogen in about 15 min.

Some tips for the first-time builders:

- Use safety goggles while drilling or cutting to protect your eyes from plastic shavings.
- Wear protective gloves when applying epoxy and handling gas connections and ensure good ventilation in the workspace.
- Ensure all connections are tight and free of debris before purging the glove box to maintain a safe, inert atmosphere.
- After assembling the glove box, perform a final leak test using diluted liquid soap before starting any sensitive experiments.
- When operating the gas cylinder, always secure the regulator properly and open the cylinder valve slowly to prevent a sudden rush of gas. Use a flow meter to set the desired flow rate accurately and close the cylinder valve completely when not in use to avoid unintentional gas release.
- Regularly check the condition of the gloves for signs of wear or damage and replace them if necessary to maintain safety and integrity.

Moreover, for extended use, periodic inspection and maintenance are needed to maintain the performance of the glove box. Gloves should be regularly checked for signs of wear, punctures, or material degradation, and replaced as needed to ensure a reliable inert atmosphere. The seals and epoxy joints around the arm holds, transfer chamber, and hose barbs should be inspected regularly, and re-sealed if any cracks or leaks are detected. Proper storage of the glove box when not in use, including keeping the container sealed and in a clean, dry environment, will further extend its lifespan and ensure reliable performance for next experiments.

## 5 Validation and characterization

The validation of the glove box was carried out using an OMD-507 In-line Oxygen Analyzer, which is capable of measuring oxygen concentrations from 0.1 ppm to 100 %. This high precision provides an accurate monitoring of oxygen levels within the glove box. During the validation process, 99.999 % UHP nitrogen gas (Linde Canada Inc.) was continuously purged into the glove box at a flow rate of about 4.7 L per minute. For further analysis of the environmental conditions inside the glove box, a battery powered digital pinless moisture meter (Mastercraft) with an accuracy of ± 4 % was used to monitor moisture levels. This device allows for non-destructive measurement of moisture content.

**Fig. 10:**
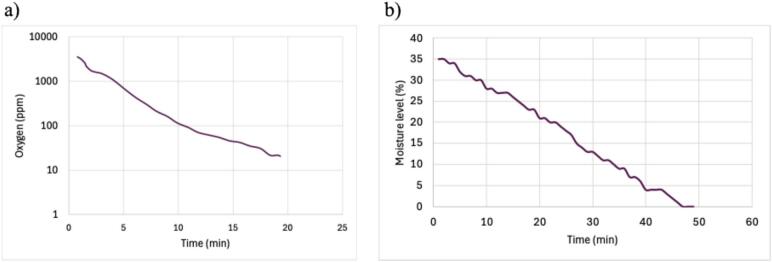
a) Oxygen reduction in the glove box vs time, b) Moisture level reduction in the glove box vs time.

The results of the validation test show that the initial oxygen concentration was reduced from 3,520 ppm to 19 ppm within 20 min as shown in Fig. 10a. Achieving such low oxygen levels is important for maintaining an inert atmosphere in applications that are related to work with air-sensitive and moisture-sensitive substances such as synthesis of tin oxide sheets (10–100 ppm), or analysis of the organic solar cells (<20 ppm) [5,7,8]. In addition, the moisture level went down to 0 % (Fig. 10b) [47]. The validation experiment was repeated with the same results.

This comprehensive validation confirms that the glove box can maintain a controlled atmsphere with small oxygen and moisture levels. Such conditions are essential for a wide range of sensitive applications, including material science research and electronics manufacturing. The amount of oxygen inside the box after purging nitrogen was measured using an oxygen analyzer. The oxygen level reduction experiments were repeated two times. For every run It took about 20 min to reduce the amount of oxygen from about 3520 ppm to <20 ppm (see supplemental data [47]). The oxygen level reduction experiments were repeated two times, and in both trials, the glove box consistently reached oxygen concentrations of 19 ppm within 20 min, which confirms reliable performance under standard operating conditions. It should be pointed out, however, fluctuations in oxygen levels can occur if the glove box is frequently opened or if minor leaks develop over time.

Additionally, while the current system does not include integrated sensors for continuous monitoring, such features are discussed as potential future improvements.

Compared to existing DIY glove box designs, which often lack a transfer chamber and have not been validated for low oxygen levels, the presented open-source glove box offers a validated inert atmosphere (reduction to 19 ppm) and includes a customizable transfer chamber that allows safe loading and unloading of materials without compromising the internal environment. This design also leverages digital distributed manufacturing via 3D printing, which offers customization not found in conventional DIY setups. In contrast to commercial glove boxes, which can cost up to CAD$15,120, this open-source design achieves over 95 % cost savings while retaining essential functionality and flexibility for laboratory applications.

While the glove box achieves a significant reduction in oxygen concentration (from 3520 ppm to <20 ppm in 20 min), the time required for purging will vary depending on the size of the box selected and initial oxygen levels. Additionally, for very sensitive applications that require ultra-low oxygen levels (<1 ppm), such as battery assembly (<0.1 ppm) [51], and cell culture experiments (<9.1 ppm), the system would need further optimization. Although iron powder was employed as an oxygen scavenger, it did not reduce oxygen levels below 19 ppm, which indicates the need for more efficient scavenging materials or methods. Also, the moisture levels were measured using a moisture meter with an accuracy of ±4 %. This accuracy may be low for applications that require extremely dry environments. It should be noted that the sensors used in this work for oxygen and moisture validation were third-party devices not integrated directly into the glove box itself. Many commercial glove boxes incorporate integrated sensors for real-time monitoring, which can be essential for ultra-sensitive applications requiring continuous atmosphere control. Although this open-source design does not include built-in sensors, future developments could explore the incorporation of integrated sensors for continuous monitoring, to provide enhanced control over experimental conditions.

A limitation of this work is the inherently porous nature of the untreated printed parts produced using FFF-based 3D printing, which may affect long-term sealing performance. To mitigate this limitation, a high infill density (100 %) can be employed during the 3D printing process to ensure less porous structures. Although the components performed well in the performed experiments, post-treatment options such as chemical sealing with appropriate solvents [48], thermal post processing [52], the use of atomic layer deposition (ALD) [[53], [54], [55], [56]], electroless plating [57] or epoxy coating, photo resins or other coatings [[58], [59], [60]] are recommended to enhance gas impermeability and maintain low oxygen concentrations during extended use or in more sensitive applications. An advantage of this open-source and 3D-printed approach, however, is that any component that becomes unsuitable for use over time can be easily reprinted and replaced, which minimizes operational disruptions and reducing costs.

Future developments could explore more effective oxygen scavengers that are capable of reducing oxygen levels to below 1 ppm and would enable the glove box to be used in more sensitive applications, such as activated carbon [61], molecular sieves [62], and copper-based scavengers [63]. Also, adding automated control systems that adjust purging rates in real-time based on oxygen and moisture sensor feedback could further enhance efficiency. By reducing the reliance on constant purging and improving the overall efficiency of gas use, future versions of the glove box could contribute to sustainability and reduced operational costs, particularly in research and industrial applications that require long-term use of inert environments.

## Declaration of competing interest

The authors declare that they have no known competing financial interests or personal relationships that could have appeared to influence the work reported in this paper.
